# Prenatal Exposure to Phthalates and Anogenital Distance in Male Infants from a Low-Exposed Danish Cohort (2010–2012)

**DOI:** 10.1289/ehp.1509870

**Published:** 2015-12-15

**Authors:** Tina Kold Jensen, Hanne Frederiksen, Henriette Boye Kyhl, Tina Harmer Lassen, Shanna H. Swan, Carl-Gustaf Bornehag, Niels E. Skakkebaek, Katharina M. Main, Dorte Vesterholm Lind, Steffen Husby, Anna-Maria Andersson

**Affiliations:** 1 Department of Environmental Medicine, Institute of Public Health, University of Southern Denmark, Odense, Denmark; 2 Odense University Hospital, Hans Christian Andersen Children’s Hospital, Odense, Denmark; 3 Department of Growth and Reproduction, Rigshospitalet, Copenhagen University Hospital, Copenhagen, Denmark; 4 Department of Preventive Medicine, Mount Sinai School of Medicine, New York, New York, USA; 5 Department of Health Sciences, Karlstad University, Karlstad, Sweden

## Abstract

**Background:**

Phthalates comprise a large class of chemicals used in a variety of consumer products. Several have anti-androgenic properties, and in rodents prenatal exposure has been associated with reduced anogenital distance (AGD)—the distance from the anus to the genitals in male offspring. Few human studies have been conducted, but associations between the anti-androgenic phthalates and male AGD have been reported.

**Objective:**

We aimed to study the association between phthalate exposure in late pregnancy in Danish women pregnant in 2010–2012 and AGD in their male infants at 3 months of age (n = 273).

**Methods:**

In the Odense child cohort study, urinary concentrations of 12 phthalate metabolites of diethyl, di-n-butyl, diisobutyl, di(2-ethylhexyl), butylbenzyl, and diisononyl phthalate (DEP, DnBP, DiBP, DEHP, BBzP, and DiNP, respectively) were measured among 245 mothers of boys at approximately gestational week 28 (range, 20.4–30.4) and adjusted for osmolality. AGD, penile width, and weight were measured 3 months after the expected date of birth. Associations between prenatal phthalate and AGD and penile width were estimated using multivariable linear regression adjusting for age and weight-for-age standard deviation score.

**Results:**

Phthalate levels were lower in this population than in a recent Swedish study in which phthalates were measured in the first trimester. No consistent associations were seen between any prenatal phthalate and AGD or penile width. Most associations were negative for exposures above the first quartile, and for ln-transformed exposures modeled as continuous variables, but there were no consistent dose–response patterns, and associations were not statistically significant (p > 0.05).

**Conclusion:**

We found no significant trends towards shorter AGD in boys with higher phthalates exposures in this low exposed Danish population.

**Citation:**

Jensen TK, Frederiksen H, Kyhl HB, Lassen TH, Swan SH, Bornehag CG, Skakkebaek NE, Main KM, Lind DV, Husby S, Andersson AM. 2016. Prenatal exposure to phthalates and anogenital distance in male infants from a low-exposed Danish cohort (2010–2012). Environ Health Perspect 124:1107–1113; http://dx.doi.org/10.1289/ehp.1509870

## Introduction

Phthalates are used as plasticizers in soft polyvinyl chloride (PVC) and found in a large number of commonly used consumer products including food, building materials, plastics, cosmetics, cleaning products, packages, and toys (Bornehag et al. 2005; Rudel et al. 2011). They are found in indoor air (Bergh et al. 2012), dust (Langer et al. 2014), food, and drinking water (Shi et al. 2012), and humans are exposed through multiple routes. Phthalates are present in urine (Frederiksen et al. 2014), blood (Frederiksen et al. 2010), and breast milk (Fromme et al. 2011) and cross the placental barrier (Jensen et al. 2012). A recent state of the art report from the World Health Organization provides new evidence for several human health risks from exposure to phthalates and other endocrine disruptors (EDCs) including cancer, metabolic outcomes (e.g., overweight and obesity), asthma and allergy, neurodevelopmental outcomes and behavior, as well as reproductive health and sexual development (Bergman et al. 2013).

Anogenital distance (AGD; distance from anus to genitals) is routinely used in animal toxicology studies and is sensitive to anti-androgenic exposure. In rodents AGD has been shown to reflect the amount of androgen to which a male fetus is exposed in early development; males have longer AGD than females, and higher *in utero* androgen exposure results in longer AGD. Numerous studies have shown that prenatal phthalate exposure—notably DEHP [di(2-ethylhexyl) phthalate], DnBP (di-*n*-butyl phthalate), DiBP (diisobutyl phthalate), BBzP (butylbenzyl phthalate)—shortens male AGD in rodents (Foster 2006; Saillenfait et al. 2008). For DiNP animal data are much more limited, but shortened male AGD has also been reported following prenatal exposure to this phthalate (Boberg et al. 2011; Clewell et al. 2013; Lee et al. 2006).

Few human studies have been conducted. The first American study among 134 mother–son pairs reported significant association between maternal exposure to several phthalates measured in urine and reduced AGD in the male offspring (Swan et al. 2005), and a later publication found an inverse association between maternal urine DEHP exposure and AGD and penile size (Swan 2008). Similar findings were reported in smaller Japanese and Mexican studies (Bustamante-Montes et al. 2013; Suzuki et al. 2012). However, a small study (*n* = 33) from Taiwan could not confirm the findings (Huang et al. 2009). A recent Swedish study found inverse association between maternal urinary DiNP metabolites and AGD, which is noteworthy given the recent substitution of DiNP for DEHP (Bornehag et al. 2014), whereas a new U.S. study found association with the DEHP metabolites MEHP [mono(2-ethylhexyl) phthalate], MEHHP [mono(2-ethyl-5-hydroxyhexyl) phthalate] and MEOHP [mono(2-ethyl-5-oxohexyl) phthalate] and AGD (Swan et al. 2015).

We therefore prospectively investigated the association between maternal urinary phthalate metabolite concentrations in pregnancy and AGD and penile width in the male offspring in a sample of 273 mother–son pairs in the Odense Child Cohort study.

## Methods

### Study Settings and Design

The study was based on data from the Odense Child Cohort (Kyhl et al. 2015). Briefly, newly pregnant women residing in Odense, Denmark, between 2010 and 2012 were recruited at a voluntary information meeting about ultrasound examinations, at first antenatal midwife visit, or at the ultrasound examination at Odense University Hospital at gestational age (GA) 8–16 weeks. Odense University Hospital is the only hospital in the municipality. Last menstrual period was used to calculate the GA (in weeks) of all participants. Of the eligible population of 6,707 pregnant women, 4,017 women were informed about the study and 2,874 (42.9%) enrolled in the cohort; 2,500 live births are being followed up at 3 and 5 years of age now. Inclusion criteria were living in the municipality of Odense and giving birth there. Participants were better educated and more often of Danish origin than nonparticipants (Kyhl et al. 2015). Fasting spot urine samples were collected at approximately GA 28 weeks before 0930 hours and stored in freezers at the Odense Patient data Explorative Network (OPEN).

### Phthalate Measurements

Participants provided a urine sample around week 28 of gestation (median 28.7 weeks, range 26.4–30.4 weeks of gestation). Samples were stored at –80°C until chemical analyses. Phthalate metabolite concentrations were measured in a subset of 565 women, and among these women, 293 women gave birth to live-born, singleton boys. We excluded women of non-Caucasian origin (*n* = 15) and those with missing information on ethnicity (*n* = 5), leaving 273 pairs of boys and their mothers eligible for analyses.

Urine samples were analyzed for total content (free and conjugated) of 12 phthalate metabolites: monoethyl phthalate (MEP), mono-*n*-butyl phthalate (MnBP), mono-iso-butyl phthalate (MiBP), monobenzyl phthalate (MBzP), MEHP, MEHHP, MEOHP, mono(2-ethyl-5-carboxypentyl) phthalate (MECPP), mono-iso-nonyl phthalate (MiNP), mono-hydroxy-iso-nonyl phthalate (MHiNP), mono-oxo-iso-nonyl phthalate (MHiOP) and mono-carboxy-iso-octyl phthalate (MCiOP) by liquid chromatography–tandem mass spectrometry (LC-MS/MS) with preceding enzymatic deconjugation followed by solid phase extraction. The method for preparation of samples, standard solutions, and quality controls as well as the instrumental analysis and general method validation has previously been described in detail (Frederiksen et al. 2010). The Chemical Laboratory at Department of Growth and Reproduction, Copenhagen University Hospital, served as reference laboratory for analysis of phthalate metabolites in a European biomonitoring project (http://www.eu-hbm.info/cophes) and further participates yearly in the German External Quality Assessment Scheme program (G-EQUAS).

Urinary osmolality, which is a measure of urinary dilution, was measured by the freezing point depression method using automatic cryoscopic osmometer (Osmomat® 030; Gonotec, Berlin, Germany). For each nine samples, a control standard urine pool was measured. Mean urinary osmolality for this standard pool (*n* = 77) was 0.825 Osm/kg with a relative standard deviation (RSD) of 1.85%. The median (range) osmolality of all urine samples included in this study was 0.63 (0.123–1.117) Osm/kg. We used urinary osmolality to adjust for urinary dilution. In contrast to urinary creatinine adjustment, which has the limitation that urinary creatinine excretion varies with sex, age, body mass index (BMI), fat-free mass, and even ethnicity, and urinary specific gravity (which is not only influenced by the number of molecules in urine but also by their molecular weight and size), urine osmolality is directly related to the number of particles in solution and is unaffected by the molecular weight and size of these particles (Frederiksen et al. 2013). In subjects with normal renal function, osmolality thus reflects an individual’s hydration status.

A total of 565 samples were analyzed: 196 samples from September 2011 through January 2012 (of whom 98 gave birth to a boy) (Tefre de Renzy-Martin et al. 2014) and 369 samples from December 2012 through January 2013 (of whom 195 gave birth to a boy) (Frederiksen et al. 2014). The first 196 samples were selected randomly, whereas the last 369 were selected based on the availability of information from questionnaires, birth records and clinical examination of the child at 3 months. We observed higher levels of some phthalate metabolites in the first subset (*n* = 196, especially DiBP metabolites) compared with the second subset of samples (*n* = 369). Therefore, 20 samples from each of the first and second subset were reanalyzed in the same batch. Similar results were obtained by the reanalysis with a variation < 5% between the original and the reanalyzed samples.

### AGD Measurements

Three months after the expected date of birth, regardless of actual gestational age at birth, the children were invited to a clinical examination, which included measurements of length, weight, and AGD on 1,659 children, of whom 565 mothers had phthalates measured in urine, 273 mothers to boys. Two different measures of AGD were made using a Vernier caliper; the shorter AGD measurement was from the center of anus to the posterior base of scrotum (AGDas) and the longer from the center of anus to the cephalad insertion of the penis (AGDap) measured in mm. Penile width was measured at the base of the penis using the Vernier caliper also (millimeters). In each child, all genital measures were repeated three times, and their arithmetic mean was calculated. In addition, 13 boys were measured by two examiners.

Among the 293 women who had phthalates measured in urine and gave birth to a boy, the final analyses included 245 (AGDas), 236 (AGDap) and 241 (penile width) due to missing data on AGD measurements or covariates. The coefficient of variation (CV) was 3% for all the triplicate AGD measurements. Interexaminer CV based on 13 measurements were, respectively, 4%, 3% and 4% for AGDas, AGDap, and penile width.

The women provided written consent to participate in the study which was approved by the local ethical committee. The research was conducted in accordance with principles of the Declaration of Helsinki (declaration of helsinki pdf).

### Data Analysis

Phthalate metabolite concentrations were adjusted for urinary osmolality normalized to the median osmolality of all samples (0.63 Osm/kg) to correct for urinary dilution. This was done for all samples with a measured phthalate concentration above the limit of detection (LOD) by dividing the individual urinary phthalate concentration (nanograms per milliliter) with the individual osmolality (osmolality per kilogram) of the urine sample and multiplying with the median osmolality of all samples (0.63 × Osm/kg) (Lassen et al. 2013). Urinary phthalate concentrations below the LOD were not adjusted for osmolality (numbers with levels below the LOD can been seen in Table 1), but substituted by the phthalate specific LOD divided by the square root of 2. To simplify the statistical analysis of all DEHP and DiNP metabolites, these were summed by addition of the molar sum of the DEHP metabolites (sum MEHP + MEHHP + MEOHP + MECPP) or the DiNP metabolites (MiNP + MHiNP + MOiNP + MCiOP) and expressed as their respective parent compound (ΣDEHPm or ΣDiNPm) by multiplication with the molecular weight of DEHP or DiNP, respectively: sum of dibutyl phthalate (DBP) isomers [ΣDBP(i + *n*)] and sums of DEHP and DiNP metabolites (ΣDEHPm and ΣDiNPm). Furthermore, because the two isomers of dibutyl phthalate are shown to be highly correlated, their metabolites (MnBP and MiBP) were summed [ΣMBP_(_ *_i_*_+_ *_n_*_)_] in this study for additional analysis (Frederiksen et al. 2010).

**Table 1:** Phthalate metabolites [ng/mL_(Osm)_] measured in gestational week 28 in 273 pregnant Danish women (2010–2012).

Diether phthalate	Phthalate metabolite	LOD	% > LOD	Mean	Minimum	Percentiles					Maximum
						5	25	50	75	95	
DEP	MEP	0.53	100	102.9	< LOD^*a*^	2.7	7.2	17.3	54.4	391.7	5380.9
DiBP	MiBP	1.10	100	38.5	< LOD^*a*^	3.7	13.3	27.1	48.2	106.7	371.1
DnBP	MnBP	1.43	96	17.8	< LOD	1.5	6.0	12.5	23.0	51.2	184.3
	∑MBP_(*i* + *n*)_			56.4	< LOD	5.6	20.4	40.4	75.9	167.8	424.5
BBzP	MBzP	1.14	69	6.5	< LOD		< LOD	2.6	5.9	16.2	546.0
DEHP	MEHP	0.14	89	2.0	< LOD	< LOD	0.4	1.2	2.3	5.5	61.1
	MEHHP	0.91	91	7.4	< LOD	< LOD	2.4	5.2	9.1	19.4	120.8
	MEOHP	0.67	93	5.9	< LOD	< LOD	2.2	4.4	7.1	14.8	106.4
	MECPP	0.55	97	6.9	< LOD	0.8	2.7	5.4	8.7	18.6	88.2
	∑DEHPm			29.3	< LOD	1.4	11.4	21.7	36.1	73.0	500.4
DiNP	MiNP	0.61	12	0.2	< LOD				< LOD	1.2	11.9
	MHiNP	0.26	90	5.4	< LOD	< LOD	0.7	1.7	4.1	14.2	336.4
	MOiNP	0.25	86	2.6	< LOD	< LOD	0.4	1.2	2.9	9.2	46.9
	MCiOP	0.11	100	9.1	< LOD^*a*^	0.5	2.0	3.9	9.3	24.7	196.4
	∑DiNPm			22.9	< LOD	0.7	4.3	9.0	22.5	67.3	476.1
∑MBP_(i + n)_, sum of MiBP and MnBP. ∑DEHPm, molar sum of DEHP metabolites expressed as excreted DEHP (ng/mL). ∑DiNPm, molar sum of DiNP metabolites expressed as excreted DiNP (ng/mL). ^***a***^Less than 1% were below LOD.											

Osmolality-adjusted phthalate metabolites [nanograms per milliliter_(osm)_] were divided into quartiles based on the distribution among the 273 women (MBzP was divided as levels below and above the median because 31% were < LOD). These metabolites were also entered in the statistical model as a continuous variable transformed by use of the natural logarithm. The AGD measurements and the penile width were left untransformed due to acceptable normal distributions of the residuals after visual inspection of histograms and normal probability plots. We calculated the distribution of ADG as well as the correlations (Spearman correlation coefficients) between the genital measures. Differences in distributions of phthalate concentrations according to population characteristics were assessed by Kruskal–Wallis test. Multivariable linear regression analysis was then used to analyze the associations between urinary phthalate excretion and AGD measurements and penile width adjusted for potential confounders. Confounders adjusted for in the models were factors associated with phthalate concentrations and AGD and or penile width. AGD measurements vary with age and weight of the child, and because the clinical examination was scheduled to take place 3 months after expected date of birth we constructed a measure of postconceptional age defined as the sum of gestational age at birth (days) and the age of the child at the AGD measurements (days). Analyses of associations between phthalate and AGD were thus adjusted for the postconceptional age and individual weight-for-age standard deviation score (*z*-score) (Swan 2008) calculated by use of Danish longitudinal growth data (Tinggaard et al. 2014). We tested trends across quartiles of phthalate exposure by inserting ordinal categorical variable coded using integer values (0, 1, 2, 3) in the regression. Also, we performed the analyses separately among women with phthalates measured in 2011–2012 and 2012–2013.

We evaluated the fit of the regression models by inspecting the residual plots for model assumption of homogeneity of variances. SPSS statistics v.19 was used and the results are presented with 95% confidence intervals (CIs); *p*-values < 0.05 were considered significant.

## Results

The 565 women with phthalate measurements were similar to other women in the cohort who delivered singletons with respect to birth weight, gestational age at delivery, child sex, maternal parity, and age. There were fewer smokers, although this was not statistically significant (3.4% vs. 5.0%; data not shown). The mean age of the women was 30.9 years at birth, 57% of the women were nulliparous, and 3.4% of the women smoked during pregnancy. A total of 9.6% of the mothers reported having had infertility treatment; the two AGDs in their boys did not differ significantly from those of women who did not report treatment (data not shown). The boys were examined at a median (range) age of 3.3 months (2.3–6.2). Median AGDas, AGDap and penile width were 36.9 mm (19.4–50.6 mm), 70.2 mm (49.1–86.2 mm), and 13.8 mm (10.3–17.4 mm). Correlation coefficients between the two different AGD measures were *r* = 0.63 (*p* < 0.0001). Penile width was weakly, though significantly, correlated with AGDas (*r* = 0.22, *p* < 0.001) and AGDap (*r* = 0.14, *p* = 0.03).

All urine samples contained MEP, MiBP, or at least one of the DiNP metabolites in concentrations above the LOD, which means that all women have been exposed to, respectively, DEP, DiBP, and DiNP, and 97%, 96%, and 69% had detectable levels of, respectively, DEHP, DnBP, and BBzP (Table 1). MiBP was observed in highest concentration (median = 27.1 ng/mL) followed by MEP and MnBP (Table 1). Urine samples were collected across the year, and seasonal variation in phthalate metabolites was found but with no consistent pattern; some metabolites were higher in spring and summer whereas others were higher in autumn and winter (data not shown).

The analyses were performed with MEP, MiBP, MnBP, ΣMBP_(_ *_i_*_+_ *_n_*_)_, MBzP, ΣDiNPm and ΣDEHPm. ΣMBP_(_ *_i_*_+_ *_n_*_)_, ΣDiNPm, and ΣDEHPm were higher in women with high or low BMI and among women who smoked (Table 2). The analyses were therefore initially adjusted for smoking and BMI, which changed the estimates < 10% (data not shown). Adjustment for parity did also changed the estimates < 10%.

**Table 2:** Median maternal osmolality adjusted urinary phthalate excretion of ∑MBP_(_ *_i_*_ +_ *_n_*_)_, ∑DiNPm, and ∑DEHPm [ng/mL_(osm)_] according to maternal characteristics.

Maternal characteristics	*n*^*a*^	∑MBP_(*i* + *n*)_	∑DEHPm	∑DiNPm
All	267
Maternal age at delivery (years)
< 25	29	43.1	23.6	8.9
25–29	92	40.3	21.2	10.6
30–34	90	43.4	21.1	7.6
≥ 35	56	48.3	26.1	12.7
Prepregnancy BMI (kg/m^2^)
< 20	26	44.7*	24.0*	7.0*
20–25	142	38.8*	21.4*	8.6*
≥ 25	99	51.1*	25.7*	12.6*
Parity
Nulliparous	154	44.2*	22.0	9.0
Multiparous	113	42.8*	22.2	10.9
Maternal smoking during pregnancy
Yes	9	110.4*	44.7*	19.0*
No	258	43.0*	21.6*	9.2*
Preterm birth (before week 37)
Yes	9	43.5	20.4	19.0
No	258	43.7	22.2	9.4
Birth weight (g)
< 2,500	6	43.5	20.0	18.1
2,500–3,999	200	44.1	22.9	9.2
≥ 4,000	61	41.2	21.1	9.8
∑MBP_(i + n)_, sum of MiBP and MnBP. ∑DEHPm, molar sum of DEHP metabolites expressed as excreted DEHP (ng/mL). ∑DiNPm, molar sum of DiNP metabolites expressed as excreted DiNP (ng/mL). ^***a***^267 participants included in the table due to missing data. *p < 0.05, Kruskal–Wallis test.				

No dose-dependent association between any phthalate metabolites and AGD or penile width was found either in unadjusted or in adjusted analyses (Table 3), as observed by the lack of monotonic patterns or significant trends, or in the model for the continuous exposures. Interestingly, however, almost all estimates were negative, suggesting that AGD and penile width were shorter in boys with exposures above the first quartile than in boys with exposures below the 25th percentile.

**Table 3:** Association between short AGD (AGDas), long AGD (AGDap), and penile width in boys and quartiles of osmolality-adjusted concentrations of or continuous ln-transformed phthalate metabolites in prenatal urine expressed as a β-coefficient (expressing the change in mm) (95% CI) from an adjusted*^a^* linear regression model.

Phthalate metabolite [ng/mL_(osm)_] (quartile)	AGDas (*n* = 245)	AGDap (*n* = 236)	Penile width (*n* = 241)
MEP
1st (LOD–8.9)	Reference	Reference	Reference
2nd (9.0–19.9)	–0.64 (–2.52, 1.23)	–0.33 (–2.37, 1.72)	–0.08 (–0.49, 0.43)
3rd (20.0–54.9)	–1.68 (–3.56, 0.20)	–0.24 (–2.32, 1.85)	–0.28 (–0.70, 0.13)
4th (≥ 55)	–1.37 (–3.27, 0.54)	–0.96 (–3.01, 1.15)	–0.07 (–0.49, 0.34)
*p*-Trend^*b*^	0.09	0.41	0.52
Continuous^*c*^	–0.34 (–0.84, 0.17)	0.02 (–0.54, 0.58)	0.03 (–0.09, 0.14)
MiBP
1st (LOD–16.9)	Reference	Reference	Reference
2nd (17.0–29.9)	–0.68 (–2.58, 1.22)	0.14 (–1.93, 2.21)	–0.29 (–0.70, 0.13)
3rd (30.0–49.9)	–0.66 (–2.49, 1.18)	–0.92 (–2.93, 1.09)	–0.35 (–0.75, 0.05)
4th (≥ 55)	–0.55 (–2.49, 1.39)	–0.88 (–2.99, 1.22)	–0.25 (–0.67, 0.16)
*p*-Trend^*b*^	0.57	0.27	0.19
Continuous^*c*^	–0.03 (–0.90, 0.83)	–0.11 (–1.07, 0.84)	–0.13 (–0.32, 0.05)
MnBP
1st (LOD–7.9)	Reference	Reference	Reference
2nd (8.0–13.9)	1.18 (–0.73, 3.01)	0.19 (–1.88, 2.27)	–0.22 (–0.63, 0.20)
3rd (14.0–20.9)	–1.07 (–2.89, 0.76)	–1.37 (–3.36, 0.63)	–0.31 (–0.71, 0.08)
4th (≥ 55)	0.19 (–1.66, 2.04)	–0.07 (–2.14, 2.00)	–0.19 (–0.60, 0.22)
*p*-Trend^*b*^	0.60	0.56	0.28
Continuous^*c*^	–0.13 (–0.89, 0.63)	–0.27 (–1.10, 0.56)	–0.04 (–0.21, 0.12)
MBzP^*d*^
1st (LOD–2.49)	Reference	Reference	Reference
2nd (≥ 2.5)	–0.80 (–2.13, 0.54)	–0.72 (–2.19, 0.75)	0.06 (–0.24, 0.35)
*p*-Trend^*b*^	0.24	0.34	0.70
Continuous^*c*^	–0.42 (–1.06, 0.21)	–0.27 (–0.97, 0.43)	–0.03 (–0.17, 0.11)
∑MBP_(*i* + *n*)_
1st (LOD–24.9)	Reference	Reference	Reference
2nd (25.0–43.9)	0.44 (–1.45, 2.32)	0.32 (–1.74, 2.37)	–0.53 (–0.94, –0.12)
3rd (44.0–69.9)	–0.95 (–2.89, 0.98)	–0.97 (–3.08, 1.14)	–0.40 (–0.82, 0.02)
4th (≥ 70)	–0.11 (–2.04, 1.82)	–0.69 (–2.82, 1.43)	–0.41 (–0.83, 0.00)
*p*-Trend^*b*^	0.56	0.31	0.12
Continuous^*c*^	–0.08 (–0.94, 0.78)	–0.16 (–1.10, 0.77)	–0.10 (–0.29, 0.08)
∑DiNPm
1st (LOD–4.9)	Reference	Reference	Reference
2nd (5.0–9.9)	–0.88 (–2.75, 0.98)	–0.21 (–2.27, 1.86)	–0.07 (–0.48, 0.33)
3rd (10.0–19.9)	–1.24 (–3.21, 0.73)	0.08 (–2.06, 2.22)	–0.29 (–0.72, 0.14)
4th (≥ 20)	–0.29 (–2.17, 1.59)	0.99 (–1.05, 3.03)	0.05 (–0.36, 0.45)
*p*-Trend^*b*^	0.74	0.31	0.98
Continuous^*c*^	–0.15 (–0.75, 0.44)	0.22 (–0.42, 0.87)	–0.02 (–0.15, 0.11)
∑DEHPm
1st (LOD–13.9)	Reference	Reference	Reference
2nd (14.0–21.9)	–1.05 (–2.95, 0.85)	–1.17 (–3.26, 0.93)	0.06 (–0.36, 0.48)
3rd (22.0–33.9)	–1.25 (–3.17, 0.67)	–0.51 (–2.61, 1.59)	0.09 (–0.33, 0.51)
4th (≥ 34)	–1.16 (–3.08, 0.77)	–0.45 (–2.56, 1.66)	0.04 (–0.39, 0.46)
*p*-Trend^*b*^	0.25	0.86	0.84
Continuous^*c*^	–0.47 (–1.35, 0.41)	–0.33 (–1.29, 0.63)	0.07 (–0.13, 0.26)
^***a***^Adjusted for the postconceptional age (defined as the sum of gestational age at birth and the age of the child at the AGD measurements) and individual weight for age standard deviation score (*z*-score). ^***b***^*p*-Value for trend across quartiles of phthalate exposure inserting ordinal categorical variable. ^***c***^Ln-transformed osmolality adjusted phthalate concentration. ^***d***^MBzP were divided as levels below and above medians because 31% were < LOD.			

Mean AGDas was lower for boys in the second, third, and fourth quartiles of MEP compared with boys in the first quartile [–0.64 mm (95% CI: –2.52, 1.23 mm), –1.68 mm (95% CI: –3.56, 0.20 mm), and –1.37 mm (95% CI: –3.27, 0.54 mm), respectively], though differences were not statistically significant (Table 3). Maternal ΣDiNPm and ΣDEHPm levels in the second, third, and fourth quartiles were associated with shorter AGD in the boys compared with levels in the first quartile. Exposure levels in the third quartile were associated with shorter AGDas than in the fourth quartile [–1.24 (–3.21, 0.73) vs. –0.29 (–2.17, 1.59) and –1.25 (–3.17, 0.67) vs. –1.16 (–3.08, 0.77)].

## Discussion

In this prospective study among 245 mother–son pairs, no dose–response association between maternal phthalate exposure and AGD or penile width in the male offspring was found. The exposure levels before adjustment in our cohort’s women were lower than in some previous studies (Table 4). Interestingly, median concentrations of all phthalate metabolites were about two to four times lower in our study than were levels observed in a recent Swedish study (Bornehag et al. 2014) in which both laboratories participated in the same quality-control program project, and the Danish laboratory served as reference laboratory (Schindler et al. 2014). Compared with the American study of future families from 1999 through 2002 the Danish levels were also lower for DEP, BBzP, and DEHP but higher for both DiBP and DnBP (Swan et al. 2005). A new U.S. study found higher levels of DEP and DEHP metabolites, but lower levels of DBP metabolites compared with our study (Swan et al. 2015) (Table 4), whereas the levels in three smaller studies were comparable with ours. In addition, except for the MBzP metabolites, all phthalate metabolites were measurable in > 95% of the samples in our study, the Swedish study and the two U.S. studies. In the U.S. and Swedish studies, the women delivered urine samples in first trimester, whereas we collected urine samples in second and third trimester. There is limited information about changes in phthalates over the course of pregnancy, but two studies have reported decreases in DEHP levels during pregnancy (Braun et al. 2012; Ferguson et al. 2014). A single spot urine sample may reasonably classify MEP and MBP concentrations during pregnancy, but more than one sample may be necessary for MBzP and DEHP (Adibi et al. 2008; Braun et al. 2012). In addition, the Swedish study collected morning urine samples, in which phthalate levels may be higher than in spot urines because phthalate levels peak 2–8 hr after intake (Frederiksen et al. 2013). Our women were fasting, however, and their samples were collected before 0930 hours, so it is unlikely that phthalate intake from breakfast would have been excreted. The differences in phthalate levels between our study and the Swedish study may be partly attributed to differences in timing of urine collection as well as differences in lifestyle and consumer behavior factors that may affect phthalate exposure.

**Table 4:** Characteristics of cohort studies measuring AGD and distribution (median and 25–75 percentiles) of unadjusted/raw urinary levels (ng/mL) of phthalate metabolites.

Study characteristics	Odense child cohort (Kyhl et al. 2015)	Study of future families (Swan et al. 2005)	SELMA study (Bornehag et al. 2014)	TIDES (Swan et al. 2015)	Japanese study (Suzuki et al. 2012)	Mexican study (Bustamante-Montes et al. 2013)	Women undergoing amniocentesis (Huang et al. 2009)
Country and year	Denmark, 2010–2012	USA, 1999–2002	Sweden, 2008–2009	USA, 2010–2012	Japan, 2005–2006	Mexico, ND	Taiwan, 2005–2006
Trimester of urine sampling	2nd and 3rd	2nd and 3rd	1st	1st	2rd	3rd	1st
Number of participants	245	134	196	753	111	73	33
AGD measurements	3 months	15 months	19–21 months	At birth	At birth	At birth	At birth
Findings	No associations	MBP ≥ AGDap↓ MEP ≥ AGDap↓ MiBP ≥ AGDap↓	DiNP metabolites ≥ AGDas↓	MEHP ≥ AGDas↓ MEOHP ≥ AGDas↓ MEHHP ≥ AGDas↓	MEHP ≥ AGDap↓	“Total phthalate” ≥ AGDap↓	No associations
β-value multivariable linear regression for positive findings	Ln MEP β = –0.34, *p* = 0.36	Log MBP, MEP, MiBP –0.59, *p* = 0.03 –0.40, *p* = 0.02 –0.77, *p* < 0.01	Log SumDiNP β = –1.69, *p* = 0.05	Log MEHP, MEOHP, MEHHP –1.12, *p* = 0.04 –1.43, *p *< 0.01 –1.28, *p* = 0.01	Log MEHP β = –0.23, *p* = 0.02	“Total phthalate” β = –0.19, *p* = 0.04
Adjustment	Weight-adjusted AGD, post-conceptional age	Weight-adjusted AGD, maternal age	Age, gestational week of sampling, weight for age, creatinine	Age, gestational age, *z*-score, time of urine collection, maternal age, study center	Smoking, gestational week, birth order, maternal age	Birth length, creatinine	Gestational age
Phthalate metabolite (ng/mL) before adjustment
MEP	17.3 (7.2–54.4)	128.4 (53.3–436.9)	60.6 (30.7–134.1)	26.0 (9.0–81.0)	7.8 (3.4–31.7)	7.6 ± 12.7^*a*^	19.1 (4.9–324.4)^*b*^
MnBP	27.1 (13.3–48.2)	13.5 (7.2–30.9)	66.0 (43.1–111.8)	7.0 (2.5–16.6)	7.0 (ND–16.6)	0.7 ± 0.5^*a*^	79.6 (28.1–232.6)^*b*^
MiBP	12.5 (6.0–23.0)	2.5 (0.7–5.1)		4.4 (1.6–10.6)
MBzP	2.6 (0–5.9)	8.3 (3.5–23.5)	15.1 (7.9–35.6)	3.1 (1.0–9.0)	3.6 (1.2–8.7)	0.6 ± 0.2^*a*^	2.5 (0–13.9)^*b*^
MEHP	1.2 (0.4–2.3)	3.3 (1.3–9.0)	3.3 (1.9–5.9)	2.0 (0.7–4.7)	3.7 (2.1–7.1)	4.0 ± 4.2^*a*^	26.3 (11.9–120.3)^*b*^
MEHHP	5.2 (2.4–9.1)	11.4 (6.0–20.1)	15.3 (8.7–22.9)	6.1 (2.4–14.0)	7.1 (4.2–13.8)
MEOHP	4.4 (2.2–7.1)	11.1 (5.1–19.0)	10.0 (5.7–15.6)	4.4 (2.0–9.9)	7.8 (4.4–13.0)
MECPP	5.4 (2.7–8.7)		14.5 (8.0–22.5)	8.6 (3.4–18.8)
∑DEHPm^*c*^	55.6 (29.2–92.4)		148.1 (84.6–220.7)	75.0 (28.2–165.0)
MHiNP	1.7 (0.7–4.1)		6.3 (2.8–14.2)
MOiNP	1.2 (0.4–2.9)		2.8 (1.3–6.2)
MCiOP	3.9 (2.0–9.3)		8.3 (5.0–16.4)	13.2 (5.0–45.2)
∑DiNPm^*d*^	21.4 (10.3–53.7)		55.9 (28.3–124.9)
Urine	Fasting, spot urine	Spot urine	Morning urine	Spot urine	Spot urine	Spot urine	Spot urine
Adjustment	Osmolality	None	Creatinine	Specific gravidity	Specific gravidity	Creatinine	Creatinine
ND, no dates found. ^***a***^Mean ± SD, MEHP, MBzP, MEP and MBP detectable in 67%, 14%, 11% and 12%. ^***b***^Median (10th–90th percentile). ^***c***^∑DEHPm, molar sum of DEHP metabolites (MEHP + MEHHP + MEOHP + MECPP) (nmol/L) in all studies. ^***d***^∑DiNPm, molar sum of DiNP metabolites (MHiNP + MOiNP + MCiOP) (nmol/L) in all studies.							

Swan et al. (2005) found associations between shorter male AGD and prenatal exposure, particularly for DEHP metabolites, in U.S. mothers recruited in 1999–2002 and among mothers recruited 2010–2012 (Swan et al. 2015), a period during which levels declined (Table 4); the Swedish study among 196 boys recruited 2009–2010 reported stronger association between AGD and DiNP than DEHP and DEP metabolites (Bornehag et al. 2014). During this 10-year period DEHP has been replaced by DiNP in soft PVC applications. Three smaller studies have been published (Table 4) (Bustamante-Montes et al. 2013; Huang et al. 2009; Suzuki et al. 2012); two found association between MEHP and “total phthalate levels” and AGD, whereas one did not find any association between prenatal phthalate exposure and AGD. However, the latter was conducted among 33 Taiwanese women with high-risk pregnancies scheduled for amniocentesis (Huang et al. 2009) and MEP exposure levels were low (as in our study).

Phthalate exposure in Denmark has declined considerably during the past 10 years (Frederiksen et al. 2014). Our findings of considerably lower phthalate levels in Danish pregnant women than in Swedish women confirms the findings from a previous European Union coordinated study (Den Hond et al. 2015).

DEHP and DiNP are known to be anti-androgens in rodent studies, although DiNP is less potent (Foster 2006; Hannas et al. 2011), and DiNP has replaced DEHP in soft PVC because of similar properties. Rats’ exposure to DiNP during gestation and perinatally has been found to increase the incidence of reproductive malformations in male offspring and to cause alterations in fetal testicular testosterone production (Borch et al. 2004). Conflicting results between prenatal exposure to DiNP and AGD in male rats have been found: Some studies found reduced AGD (Boberg et al. 2011; Clewell et al. 2013; Lee et al. 2006) whereas others found no association (Gray et al. 2000; Masutomi et al. 2004). This may be attributable to differences in exposure levels between studies.

AGD measurements are well tolerated by all subjects and quick to perform, with < 5% intra- and interexaminer reliability, and currently few known factors need to be controlled for (age and body size). AGD measurements differ considerably among studies (Bornehag et al. 2014; Bustamante-Montes et al. 2013; Huang et al. 2009; Suzuki et al. 2012; Swan et al. 2005; Thankamony et al. 2014), which may partly be explained by differences in age at examination. Some studies measured only one AGD distance whereas others measured both the AGDas and the distance to the AGDap. The AGDas is less dependent on infant size; nevertheless the U.S. study found stronger associations for AGDap (Swan et al. 2015).

AGD is a continuous measure of developmental exposure to anti-androgens in rodents (Foster 2006). Because it can be measured in all boys it is a more sensitive marker of genital development than the birth prevalence of cryptorchidism or hypospadias, which are found in < 10% of newborns, thus requiring large study populations. Shorter AGD has been associated with hypospadias and cryptorchidism in human males (Hsieh et al. 2008; Thankamony et al. 2014). In male rodents, shortened AGD persists into adulthood (Hotchkiss et al. 2004) and predicts compromised reproductive function (reduced testis size) in the mature male (Scott et al. 2008). The same link between prenatal anti-androgen exposure and adult reproductive function is suggested by cross-sectional studies among adult men, which have found associations between AGD and adult semen quality (Eisenberg et al. 2011; Mendiola et al. 2011) and serum testosterone (Eisenberg et al. 2012a). In addition, fertile men and men with obstructive azospermia (suggested to be caused by infections) have a longer AGD than infertile men and those with nonobstructive azospermia (Eisenberg et al. 2011, 2012b). This suggests that shortened AGD may be a member of the symptom complex of the testicular dysgenesis syndrome (TDS) (Thankamony et al. 2014). This is also in line with the theory that TDS symptoms result from a disturbance in the Sertoli cell and Leydig cell differentiation during fetal life, leading to impaired testosterone production and decreased virilization (Skakkebæk 2004).

Our study has several strengths: It is large and is population based because the municipality only included one hospital. However, only 42% of the eligible women participated, and 1,659 had AGD measured; also, participants were better educated than nonparticipants. Their age at delivery resembled that of pregnant women in Denmark, and the women had no knowledge of their phthalate exposure or the AGD of their child at enrollment. It is therefore unlikely to have affected their participation. In addition, we compared women across phthalate exposure, so whether they represented the general population is therefore of less importance in the study except for generalizability. We adjusted for relevant confounders, but we cannot exclude the possibility of residual confounding by other factors associated with phthalate exposure and growth measures, such as co-exposure to other environmental chemicals, lifestyle, or health behavior.

Phthalates are quickly metabolized with a urinary excretion half-life of < 24 hr (Anderson et al. 2001, 2011; Koch et al. 2012). A single spot urine sample collected around gestational week 28 may therefore not reflect fetal exposure in the sensitive developmental window; however, a single spot urine sample may reasonably classify MEP and MBP concentrations during pregnancy, but more than one sample may be necessary for MBzP and DEHP (Adibi et al. 2008; Braun et al. 2012). The women were fasting, which may contribute to the low urine phthalate levels. However, this misclassification is likely nondifferential because it is not associated with AGD and thus may understimate the effect of phthalate exposure. In addition, temporal and seasonal variation in phthalate levels in our study population was found (data not shown), though samples were collected during the whole calendar year (data not shown). Also, phthalates were measured in two different batches; however, we reassessed 20 samples, and acceptable agreement between original and later levels were found.

## Conclusions

In conclusion, in this population based study of 245 mother–son pairs we found no consistent dose–response association between maternal phthalate exposure and AGD in the offspring. Phthalate exposure was low in this population: ≤ 50% of that in a recent Swedish study and lower than in U.S. studies (except for the DnBP isomers) conducted 1999–2002 and 2010–2012. They, however, measured phthalate levels in first trimester, and the Swedish study used morning urine whereas we measured fasting spot urine in second and third trimester, which may explain some of the differences in exposure levels.
